# Dendritic cell expression of MyD88 is required for rotavirus-induced B cell activation

**DOI:** 10.1128/jvi.00653-25

**Published:** 2025-04-30

**Authors:** Sarah E. Blutt, Amber D. Miller, Margaret E. Conner

**Affiliations:** 1 Department of Molecular Virology and Microbiology, Baylor College of Medicine 189531, Houston, Texas, USA; 2 Michael E. DeBakey Veterans Affairs Medical Center 20116, Houston, Texas, USA; 3 Huffington Department of Education, Innovation, and Technology, Baylor College of Medicine 3989, Houston, Texas, USA; University of Michigan Medical School, Ann Arbor, Michigan, USA

**Keywords:** rotavirus, B cell, dendritic cell, MyD88

## Abstract

Intestinal IgA, produced by local intestinal B cells, is thought to play a major role in protection against intestinal infections. Rotavirus, a well-characterized intestinal virus, induces a rapid viral-specific intestinal IgA response that occurs in the absence of T cells. Previous work has indicated that dendritic cells facilitate the early IgA response to rotavirus. To determine whether the early Peyer's patch B cell activation associated with rotavirus infection in mice requires dendritic cells, we depleted dendritic cells and assessed B cell activation. Depletion of CD11c^+^ cells *in vivo* prior to infection resulted in a complete abrogation of Peyer's patch B cell activation. With the use of *in vitro* cell-based assays, CD11c^+^, but not T or CD11b^+^ cells, was shown to be essential for rotavirus-induced activation of B cells. Investigation of several pathways of B cell activation revealed that dendritic cell expression of MyD88 and signaling through the type I interferon receptor were critical for the ability of the virus to induce B cell activation. These findings indicate that CD11c^+^ dendritic cells can modulate B cell responses to viruses through toll-like receptor and type I interferon signaling pathways.

**IMPORTANCE:**

Dendritic cells are key mediators of immune responses in the intestine. They can capture and process rotavirus antigens and present these antigens to B cells, which produce critical IgA antibody that is essential for clearance of rotavirus infection and protection from reinfection. In the work presented here, we demonstrate that dendritic cell expression of MyD88, a key component of pattern recognition pathways, and not classical IgA pathway molecules such as BAFF and APRIL, is critical for the ability of the dendritic cell to induce the activation of B cells. Our findings emphasize the important role that dendritic cells play in initiating and regulating immune responses including T cell-independent B cell activation. A consideration of the role of dendritic cells in B cell activation and antibody production is an important feature in the development of therapeutic and preventive modalities to combat intestinal viral infections.

## INTRODUCTION

Rotavirus is a double-stranded RNA virus that infects the epithelial cells of the small intestine, resulting in gastroenteritis that can result in significant mortality (1). Studies in humans and animal models of infection have linked clearance of and protection from rotavirus infection to the induction of rotavirus-specific IgA in the gastrointestinal tract (2, 3). Mice that lack IgA (4) or B cells (2, 5) have difficulty resolving a rotavirus infection and are not protected against reinfection. In contrast, mice that lack T cells clear rotavirus infection, develop stool rotavirus-specific IgA, and are protected against reinfection (2, 6, 7). Because IgA is the predominant antibody isotype present in humans and is largely concentrated at mucosal surfaces including the gastrointestinal tract, it is an important target in mucosal vaccine design. However, the complexity of IgA induction has hindered the development of oral vaccines that protect against infection. To design better vaccines that induce an effective IgA response, we need a better and more complete understanding of the pathways that lead to pathogen-specific T cell-independent IgA induction in the gastrointestinal system.

Rotavirus induces rapid profound activation of B cells, measured by upregulation of activation marker CD69, within the Peyer's patches of rotavirus-infected mice as early as 48 hours following exposure to the virus (8, 9). This rapid activation, which occurs in the absence of T cell activation, is followed by the local production of rotavirus-specific antibody in the Peyer's patch preceding intestinal viral clearance (8), suggesting that rotavirus induces intestinal IgA responses through T cell-independent pathways. In support of this concept, mice lacking T cells have the same fold increase in Peyer's patch activated B cells as do wild-type mice (8) and produce rotavirus-specific antibody that is protective against reinfection.

Dendritic cells are recognized as playing a significant role in the induction of B cell responses and IgA production in the intestine (10). They activate B cells through traditional pathways that include CD40/CD40L (11) and the B cell receptor (12) as well as through non-canonical pathways that include BAFF/APRIL (13) and toll-like receptor signaling (14). Dendritic cells in rotavirus-infected mice were found to (1) be increased in number in the Peyer's patches at 48 hours following inoculation, (2) migrate to the sub-epithelial dome region of the Peyer's patch, (3) have increased expression of surface activation marker expression, and (4) upregulate mRNA for both pro-inflammatory and regulatory cytokines (9, 15). The activation of these dendritic cells occurs concurrently with the activation of B cells in the Peyer's patches, suggesting that dendritic cells might be an important component of the pathways through which rotavirus induces B cell responses in the Peyer's patch. Support for the relationship between dendritic cells and rotavirus-specific IgA production also arises from studies in the mesenteric lymph node (MLN) where classical BATF3-dependent dendritic cells facilitate the early IgA response to rotavirus (16), as well as from studies with human peripheral blood mononuclear cells (PBMCs) demonstrating that plasmacytoid dendritic cells are necessary and sufficient for rotavirus-induced B cell activation (17). In contrast to the MLN and PBMCs, in the spleen, both plasmacytoid and classical dendritic cells seem to contribute to B cell activation by rotavirus (18). However, the signaling pathways through which rotavirus stimulates dendritic cells to induce B cell activation have not been fully delineated.

Rotavirus-induced B cell activation can be recapitulated in an *in vitro*-based system in which single cell suspensions from immunological tissues derived from rotavirus naïve mice are incubated with rotavirus overnight (19). Here, we use this assay to examine whether several canonical and non-canonical molecules known to play a role in B cell antibody and IgA production are required for dendritic cell-mediated B cell activation. We find that dendritic cell expression of MyD88 and interferon alpha is critical for rotavirus to induce activated B cells. Both molecules shape a robust B cell response that is likely key to ensuring effective antibody production, a critical component of viral clearance and protection from reinfection.

## RESULTS

### Rotavirus-induced B cell activation in the Peyer's patches is accompanied by dendritic cell activation

**Fig 1:**
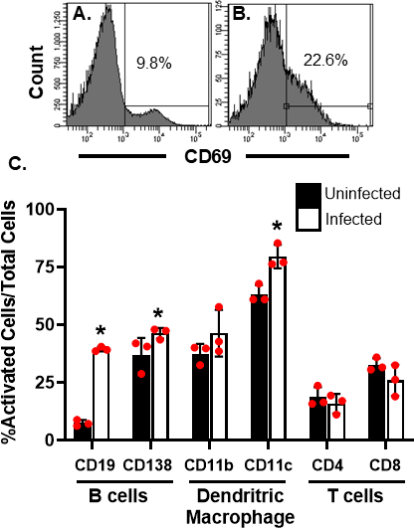
Rotavirus infection activates both B cells and dendritic cells in the murine Peyer's patch. CD-1 mice were orally inoculated with PBS (black bars) or 10^5^ ID_50_ EC_wt_ (white bars), and single cell suspensions were prepared from the Peyer's patches 48 hours later. Cells were incubated with antibodies against the indicated cell surface markers and activation marker CD69, and fluorescently labeled cells were detected and quantified using a flow cytometer. (**A, B**) Representative histograms indicating CD69 staining in total Peyer's patch cells isolated from uninfected (**A**) and infected (**B**) animals. (C) Quantification of CD69 staining in each indicated specific cell population. Each bar represents the mean number of activated cells (CD69^+^) out of the total indicated cell population (CD19, CD138, CD11b, CD11c, CD4, or CD8; percent activated) from three individual mice ± SD. *, *P* < 0.05 compared to PBS-treated animals by Mann-Whitney U. Three independent replicate experiments were performed, and a representative experiment is shown.

Rotavirus infection results in rapid and profound B cell activation characterized by increased numbers of CD19^+^/CD69^+^ B cells in the Peyer's patches of mice within 48 hours of viral exposure (8). To determine if infection activated other cell types in the Peyer's patch concurrently with the B cells, we prepared leukocyte single cell suspensions from the Peyer's patches of infected mice and assessed them for the activation marker CD69 expression by flow cytometry (Fig. 1A and B; Fig. S1). Simultaneously with the increased B cell activation (CD19^+^/CD69^+^), a smaller but still significant increase in activated B lineage plasmablast cells (CD138^+^) and CD11c^+^ cells (dendritic cells) was detected in the Peyer's patches of rotavirus-infected mice compared to uninfected mice (Fig. 1C). There was no detectable increase in activated CD4^+^ (helper T cell), CD8^+^ (cytotoxic T cell), or CD11b^+^ (macrophage) cells in Peyer's patches of infected animals compared to uninfected animals (Fig. 1C). These findings indicated that rotavirus-induced B cell activation in Peyer's patches is accompanied by a significant increase in CD11c^+^ cell activation.

### *In vivo* ablation of CD11c^+^ cells affects the early induction of Peyer's patch B cell activation

**Fig 2:**
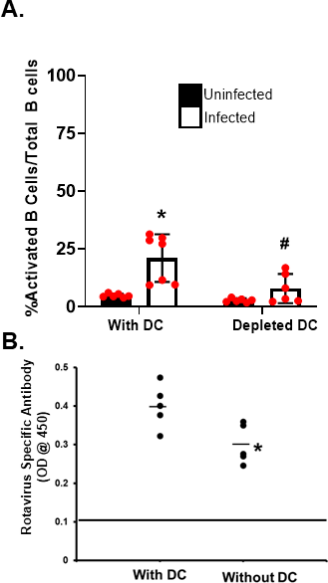
*In vivo* depletion of dendritic cells ablates Peyer's patch B cell activation following rotavirus infection. Mice expressing the diphtheria toxin receptor under control of the CD11c promoter were administered water (With DC) or diphtheria toxin (Depleted DC). Mice were inoculated with PBS (black bar) or 10^3^ ID_50_ EC_wt_ rotavirus (white bar). Four days following rotavirus exposure, the Peyer's patches were harvested from each animal. (**A**) Peyer's patches from each animal were assessed for activated B cells by flow cytometric detection of CD19 and CD69 or cultured *ex vivo* in media for 2 days. Each bar represents the mean number of activated cells (CD19^+^/CD69^+^) out of the total indicated cell population (CD19^+^; percent activated) from six to seven individual mice ± SD. *, *P* < 0.05 compared to mice receiving PBS by Mann-Whitney U. #, *P* < 0.05 compared to mice receiving rotavirus and water by Mann-Whitney U. (**B**) Peyer's patches were cultured *ex vivo* in media for 2 days followed by quantification of the amount of secreted total rotavirus-specific antibody (IgM, IgG, and IgA) measured by ELISA. Bars indicated mean level of rotavirus-specific antibody as measured by spectrophotometer of *n* = 5 individual mice. *, *P* < 0.05 compared to mice receiving PBS by Mann-Whitney U. Two independent replicate experiments were performed, and a representative experiment is shown.

To assess whether CD11c^+^ cells were important in Peyer's patch B cell activation *in vivo*, we administered diphtheria toxin to deplete CD11c^+^ cells in mice expressing the CD11c gene linked to the diphtheria toxin receptor (CD11c-DTR) (20). Flow cytometry examination of Peyer's patch single cell suspensions for B cell activation indicated that depletion of CD11c^+^ cells resulted in a significant reduction in CD69 expression on the CD19^+^ B cell population (Fig. 2A). These results indicate that CD11c^+^ cells are important for the local induction of Peyer's patch B cell activation *in vivo*. Analysis of local rotavirus-specific antibody production in the Peyer's patch via *in vitro* organ fragment culture (8) revealed a significant decrease in local antibody production (Fig. 2B). There were no differences in total numbers of B cells isolated from the Peyer's patches of DT-treated mice compared to mock-treated animals (mean: 6.1 × 10^6^, 95% confidence interval [CI]: 4.5 × 10^6^–7.9 × 10^6^ versus mean: 6.9 × 10^6^, CI: 2.4 × 10^6^–11.6 × 10^6^). These findings suggest that rotavirus-activated dendritic cells contribute to both B cell activation and antigen-specific antibody production.

### CD11c^+^ cells are required for B cell activation *in vitro*

**Fig 3:**
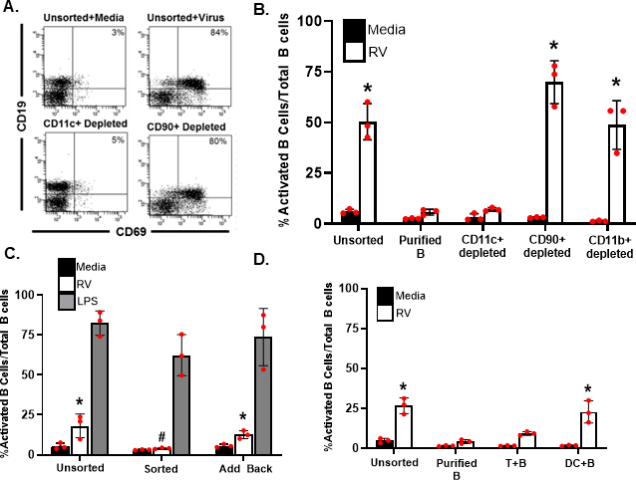
CD11c^+^ dendritic cells, but not CD90^+^ or CD11b^+^, are required for rotavirus-induced B cell activation. (**A**) Representative flow cytometry dot plots of rotavirus-treated and untreated splenic single cell suspensions isolated from CD-1 outbred mice prior to (unsorted) or following sorting (depleted). Cells were treated with rotavirus (white) or media only (black). B cell activation was assessed by expression of CD69 on the CD19^+^ population using fluorescently labeled antibodies and flow cytometry, and data are shown as mean percentage of activated B cells out of total B cells (*n* = 3) ± SD. (**B**) Undepleted splenic cell suspensions or cell suspensions depleted of all non-B cells (purified B), dendritic cells (CD11c^+^ depleted), T cells (CD90^+^ depleted), or macrophages (CD11b^+^ depleted) were treated with media (black) or rotavirus (white), and activated B cells (CD19^+^/CD69^+^) out of total B cells (CD19^+^) were quantified by flow cytometry. Each bar represents the mean number of activated B cells out of total B cells (*n* = 3 individual mice) + SD. *, *P* < 0.05 by Mann-Whitney U compared to media-treated cells. #, *P* < 0.05 by Mann-Whitney U compared to presort rotavirus-treated cells. (**C**) Splenic single cell suspensions were treated prior to selection (Unsorted) or depleted of CD11c+ (Sorted) cells prior to treatment. CD11c^+^ cells that were depleted from the cell suspensions were added back prior to treatment (Add Back). Cells suspensions were treated with media (black bar), rotavirus (white bars), or lipopolysaccharide (gray bars). Each bar represents the mean number of activated B cells out of total B cells (*n* = 3 individual mice) + SD. *, *P* < 0.05 by Mann-Whitney U compared to media-treated cells. #, *P* < 0.05 by Mann-Whitney U compared to unsorted rotavirus-treated cells. (**D**) Splenic single cell suspensions were stimulated with media (black bar) or rotavirus (white bar) prior to selection (Unsorted), following selection (Purified B), or after co-culturing with either CD4^+^ T cells (T + B) or with the Cd11c^+^ cell population (DC + B). Each bar represents the mean number of activated B cells out of total B cells (*n* = 3 individual mice) + SD. *, *P* < 0.05 by Mann-Whitney U compared to media-treated cells. #, *P* < 0.05 by Mann-Whitney U compared to unsorted rotavirus-treated cells. Three independent replicate experiments were performed, and a representative experiment is shown.

The significant reduction of Peyer's patch B cell activation following depletion of the CD11c^+^ population suggested that CD11c^+^ cells were essential for the initial B cell response to rotavirus. Previous work had suggested that both CD11c^+^ and CD11b^+^ cells could activate B cells in response to rotavirus infection (17). To assess whether CD11c^+^ cells were absolutely required for rotavirus-induced B cell activation, we depleted splenic single cell suspensions of CD11c^+^, CD11b^+^, or CD90^+^ cells (pan T cells) as a control. These cells were then treated with rotavirus or media alone, and B cell activation was assessed by flow cytometry (Fig. 3A). Total B cell populations were gated on, and the numbers of CD19^+^/CD69^+^ cells within the B cell population were quantified (Fig. S2). CD11c^+^-depleted single cell suspensions treated with rotavirus did not exhibit increases in the percentage of activated B cells out of the total numbers of B cells when compared to untreated CD11c^+^-depleted suspensions (Fig. 3B). In contrast, single cell suspensions depleted of CD90^+^ or CD11b^+^ cells and treated with rotavirus exhibited similar increases in activated B cells as did rotavirus treatment of undepleted cell suspensions (Fig. 3B). B cells alone treated with rotavirus did not exhibit significant increases in cell surface expression of CD69 when compared to untreated B cells (Fig. 3B), confirming other reports that B cell activation by rotavirus requires another cell types. To ensure that the selection process did not render the B cells unable to be activated, we compared the response of B cells treated with the B cell mitogen, lipopolysaccharide (LPS), unsorted, after sorting, and after sorting and adding back the CD11c^+^ population. LPS induced similar levels of B cell activation in all three groups (Fig. 3C), indicating that the processing and isolation did not affect the B cell capacity to respond to the virus. Adding back the depleted CD11c^+^ cells following the depletion process was associated with a recovery of rotavirus-induced B cell activation (Fig. 3C). This effect was not mouse strain dependent as three different mouse strains exhibited the same effect (Fig. S3). These findings suggest that (1) rotavirus is not a B cell mitogen and that (2) CD11c^+^ dendritic cells are sufficient to induce B cell activation following rotavirus treatment.

To determine whether the presence of CD11c^+^ cells was sufficient for rotavirus-induced B cell activation, we positively selected CD11c^+^ cells and negatively selected B cells, and the two populations co-cultured together. As a control, CD4^+^ T cells were also positively selected and co-cultured with negatively selected B cells. Only the CD11c^+^/B cell co-cultures exhibited B cell activation after virus treatment similar to what was observed in unsorted cells (Fig. 3D). Neither purified B cells nor CD4^+^/B cell co-cultures resulted in a significant increase in activated B cells after virus treatment (Fig. 3D). To assess whether the ability of CD11c^+^ cells to induce B cell activation is mouse strain specific, we examined co-cultures of CD11c^+^ cells and B cells from C57BL/6 and BALB/c mice. These co-cultures were also treated with rotavirus, and B cell activation was assessed by flow cytometry. Rotavirus induced B cell activation when B cells were cultured in the presence of CD11c^+^ cells independent of mouse strain (Fig. S3) but not when the purified B cells were cultured alone. Taken together, these results indicate that CD11c^+^, but not CD4^+^, cells are sufficient to induce B cell activation after rotavirus treatment independently of the genetic background of the cells

### A lack of MyD88 expression and interferon signaling results in defects in rotavirus-induced B cell activation

**Fig 4:**
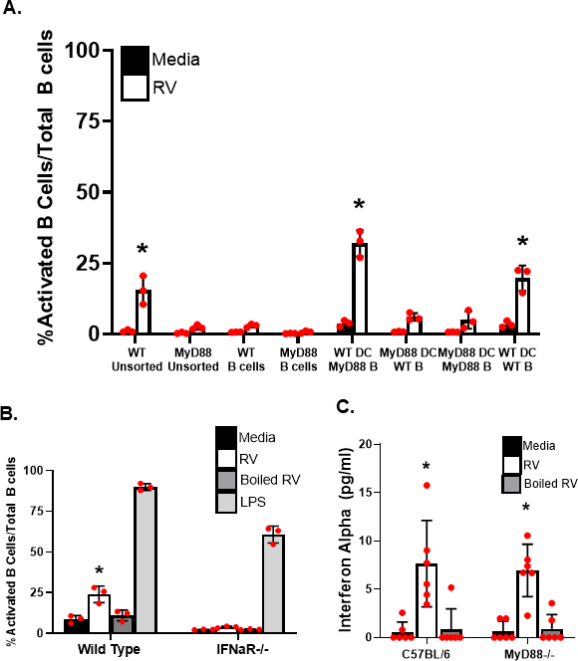
MyD88 expression in CD11c^+^ cells is required for B cell activation but not for interferon alpha induction. (**A**) Cell suspensions from MyD88^−/−^ mice or the respective WT control mice were treated with rotavirus or media control. Cell suspensions were either untouched (Unsorted); B cells were negatively selected (B cells or B) or co-cultured with positively selected WT or MyD88^−/−^ dendritic cells (DC). Percent activated B cells (CD19^+^/CD69^+^) out of total B cells (CD19^+^) were assessed. Each bar represents the mean of values obtained from three individual mice ± SD. *, *P* < 0.05 by Mann-Whitney U compared to media-treated cells. #, *P* < 0.05 by Mann-Whitney U compared to presort rotavirus-treated WT cells. (**B**) Splenic cells from the indicated knockout mice were stimulated overnight with media (black), 0.01 µg/µL purified rotavirus (RV, white), boiled rotavirus (dark gray), or 0.01 µg/µL LPS (light gray), and the percent of CD19^+^ cells that expressed CD69 out of the total CD19^+^ population was determined by flow cytometry for each mouse. Mean ± SD (*n* = 3–7) are shown. *, *P* < 0.05 compared to rotavirus-treated WT mouse. (**C**) Splenic single cell suspensions from wild type (WT) or MyD88^−/−^ (MyD88) mice were treated with either media (black bars), rotavirus (white bars), or boiled rotavirus (grey bars) for 24 hours followed by quantitation of interferon alpha in the supernatant by ELISA. Each bar represents the mean amount of interferon alpha obtained from six individual mice ± SD. *, *P* < 0.05 by Mann-Whitney U compared to media-treated cells. At least three independent replicate experiments were performed, and a representative experiment is shown.

Several pathways have been implicated in DC-mediated B cell activation by rotavirus, including BAFF/APRIL (17), type I interferon (17, 18), toll-like receptor 7 signaling (18), and TGFbeta signaling (16). To identify the potential pathway through which dendritic cells signal Peyer's patch B cell activation following rotavirus exposure, we isolated and screened leukocytes from mice lacking various proteins implicated in B cell activation pathways. These molecules include proteins important in B cell receptor-mediated signaling, traditional T cell-mediated pathways, and pathways implicated in innate B cell responses (Fig. S4). As expected, based on our current and previous findings that T cells are not important for rotavirus-induced B cell activation (Fig. 3B and D) (2, 7), we found that lack of CD40 and CD40L expression in leukocytes did not reduce rotavirus-induced B cell activation compared to their respective wild-type cells. Lack of MHCII expression also did not affect virus-induced B cell activation. Similarly, there were no defects in rotavirus-induced B cell activation compared to their respective wild-type cells in leukocytes expressing a transgenic B cell receptor (MD4, OVA-BCR) or an incompletely assembled B cell receptor (XID). We also observed that leukocytes lacking expression of proteins involved in T cell-independent B cell activation (BAFF, TACI, and APRIL) were activated following rotavirus treatment to the same levels as their respective wild-type cells. Supporting a link between rotavirus-induced B cell activation and toll-like receptor signaling, we found that lack of expression of MyD88, a protein important in toll-like receptor signaling, significantly reduced the ability of rotavirus to activate B cells within the leukocyte suspensions (Fig. 4A; Fig. S5). However, the lack of expression of TIRAP and TRIF, also important in toll-like receptor signaling, did not alter rotavirus-induced B cell activation (Fig. S5). Finally, B cells in cell suspensions that were defective in type I interferon alpha receptor expression were unable to be activated following rotavirus treatment (Fig. 4B), providing additional support for the role of type I interferon in T cell-independent activation of B cells by rotavirus (17, 18).

### CD11c^+^ expression of MyD88 is critical for B cell activation but not for interferon production

Previous work showed that myeloid dendritic cells exposed to rotavirus produced type I interferons (21) and that interferon alpha in particular is responsible for rotavirus-induced B cell activation (17, 22). Based on our findings that both MyD88 and type I interferon were important for rotavirus-induced B cell activation, we assessed whether leukocytes lacking MyD88 expression produced interferon alpha following virus treatment. Although leukocyte suspensions lacking MyD88 expression had defects in B cell activation following virus treatment (Fig. 4A; Fig. S5), the cells produced similar amounts of secreted interferon alpha into the supernatants as did wild-type cell suspensions following virus treatment (Fig. 4C) and were still capable of being activated using nonspecific agents such as phorbol myristate acetate (PMA) (Fig. S5). We previously showed that rotavirus structure, which can be destroyed by boiling, is critical for the ability to activate B cells (19). Interferon alpha production by both wild type and MyD88-deficient cell suspensions was abrogated when the virus was boiled before treatment (Fig. 4C), indicating that either viral protein structure or viral replication is important to the induction of B cell activation via MyD88. To determine whether MyD88 expression was important for B cell activation in the CD11c^+^ cell or in the B cell, we positively selected CD11c^+^ cells from either WT or MyD88^−/−^ cell suspensions. The WT CD11c^+^ cells were co-cultured with MyD88^−/−^ B cells, and MyD88^−/−^ CD11c^+^ cells were co-cultured with WT B cells. Only the WT CD11c^+^/MyD88^−/−^ B cell co-cultures demonstrated activation of the B cells following virus treatment (Fig. 4A), indicating that MyD88 expression by the CD11c^+^ cell, and not the B cell, is critical for rotavirus to induce B cell activation. Supporting a role for MyD88 in rotavirus-specific IgA production, MYD88^−/−^ mice infected with rotavirus exhibited reduced levels of both fecal and serum rotavirus-specific IgA, supporting the contribution of the MyD88-associated pathways and antigen-specific IgA antibody production (Fig. S6).

## DISCUSSION

Dendritic cells are powerful antigen-presenting cells and can produce many important factors critical in B cell activation and maturation (23). Previous work demonstrated that dendritic cells can bind to and internalize rotavirus particles (24) or proteins (25) and play a role in T cell-independent rotavirus-specific B cell activation (6, 8). To further interrogate the subtype of dendritic cell and key signaling pathways that mediate the rotavirus-specific B cell response, we utilized both *in vivo* and *in vitro* assays. *In vivo*, we find that CD11c^+^ dendritic cells are required for early Peyer's patch B cell activation and rotavirus-specific IgA production. Using a reductionist *in vitro* assay, we delineate that CD11c^+^ cells are sufficient to induce activation and that CD11c^+^ cell expression of the toll-like receptor signaling molecule MyD88 is essential for the ability of the virus to modulate B cell activation. Taken together, these results support the concept that dendritic cells play a key role in the generation of T cell-independent IgA production in the intestine during rotavirus infection through signaling that involves toll-like receptors.

Rotavirus has been shown to associate with activation of several types of cells in the immune system including macrophages and dendritic cells (9, 24, 26–32). Our finding that CD11c^+^ dendritic cells isolated from the Peyer's patches of rotavirus-infected mice had increased upregulation of CD69 supports previous findings that, following rotavirus exposure, Peyer's patch dendritic cells appear to be activated and increased in number following infection (9, 32). It also corroborates work examining the activation of CD11c^+^ splenic dendritic cells following rotavirus treatment (17). *In vitro*, CD11c^+^ dendritic cells were necessary and sufficient to induce B cell activation following rotavirus treatment. In some cases, the level of B cell activation by CD11c^+^ cells appears lower when compared to levels induced in unsorted cells, and further investigations will determine whether other cell types can augment the activation potential of the dendritic cells. One caveat in our approach may be the lack of specificity of CD11c and CD11b as markers for dendritic and macrophage subsets, respectively. CD11b is known to be expressed on some subsets of dendritic cells (33), and macrophages and some intestinal monocyte populations may express CD11c (33, 34). Further and more definitive markers will be necessary to characterize the exact phenotype of the activated CD11c^+^ population. Additionally, we did observe smaller increases in activation of both CD11b^+^ and CD138^+^ cells (Fig. 1B) that may or may not play a significant biological role in the Peyer's patch immune response to rotavirus. A more detailed study focusing on these specific cell types will ultimately demonstrate their relevance in the clearance of infection and protection from reinfection.

Dendritic cells contribute to innate and adaptive immunity through directing both T cell-dependent and independent antibody production. There are several possible signaling pathways through which the rotavirus-activated dendritic cells might modulate T cell-independent rotavirus-specific B cell responses. Using leucocyte suspensions from genetically modified mice, we interrogated multiple well-known T cell-dependent and independent pathways that are linked to IgA production. As expected, based on our previous work (6, 8) as well as that of others (2, 5, 35), molecules that were found to play a role in T cell-dependent pathways had no effect on the induction of B cell activation by rotavirus. These mice also clear an initial rotavirus infection with normal kinetics and produce rotavirus-specific fecal antibody (Fig. S4). Unexpectedly, BAFF/APRIL/TACI, which have been linked to T cell-independent IgA production in the intestine (36, 37), did not appear to be critical for rotavirus-induced B cell activation. This finding does not eliminate a role for these molecules as the assay used only measures the initiating event of IgA production (B cell activation), and they could play a critical role downstream in the generation of IgA. However, infection of mice lacking expression of BAFF, TACI, or APRIL resulted in normal clearance of rotavirus and in the production of rotavirus-specific intestinal IgA. These data provide further support that these molecules are not required for the T cell-independent antibody response to infection (Fig. S4). We cannot rule out the possibility that these molecules can all drive rotavirus-induced IgA production independently and that lack of any single molecule has no effect.

Dendritic cells have long been known to recognize pathogens via pattern recognition receptors in the toll-like receptor family. The data here support a role for toll-like receptor signaling in the T cell-independent B cell response to rotavirus. We find that the lack of expression of MyD88 on CD11c^+^ dendritic cells, an essential toll-like receptor signaling adaptor protein known to play a role in enhancing dendritic cell antigen presentation (38), results in an abrogation of rotavirus-induced B cell activation. Recent work has demonstrated that stimulation of B cells by TLR ligands results in class switch recombination (39, 40), supporting a role for this pathway in IgA production. However, other molecules that play a role in toll-like receptor signaling, TIRAP and TRIF (41, 42), were not critical. Recently, dendritic cells from TRIF^−/−^ mice were shown to have impaired T cell antigen presentation; however, the effect on B cells was not examined (43). We find that mice lacking either TIRAP or TRIF expression have no significant differences in fecal rotavirus-specific IgA levels compared to wild-type mice (Fig. S6) and, as expected based on this finding, clear rotavirus infection with normal kinetics. As expected (17), lack of interferon alpha signaling resulted in an abrogation of B cell activation by the virus; however, wild-type levels of interferon alpha were not sufficient to overcome the defect in MyD88 signaling, suggesting that interferon alpha alone is not sufficient to induce B cell activation. It may be that other ligands of the interferon alpha receptor (44) are important and may explain data suggesting that this pathway is a critical component of rotavirus-induced B cell activation.

B cells can be activated by and secrete antibody without engagement of the BCR (45). Our finding that B cells with non-mature (XID) or transgenic BCRs (MD4, OVA-BCR) are activated after rotavirus treatment provides new evidence that rotavirus may initiate signaling in B cells through non-BCR mediated pathways. This is consistent with reports that B cells internalize and present viral proteins, irrespective of their BCR specificity (46), and polyclonal BCR-independent B cell activation has been hypothesized as a mechanism of B cell memory maintenance (47–50). Together, these data implicate other molecular pathways that either augment or replace BCR signaling pathways in B cell activation and antibody responses, such as toll-like receptor signaling. Our findings suggest that B cell activation in response to a viral pathogen can occur through non-classical pathways independent of the BCR. The initiation of alternative B cell activation pathways may result from high levels of antigen that are present during an acute infection with a rapidly replicating pathogen like rotavirus that results in rapid production of high levels of antibody at the mucosal surface. This early response is crucial to combat the rapidly replicating virus and the dehydrating diarrhea that accompanies it. Likely, once antigen concentrations exceed a specific threshold, T cell-independent B cell activation is triggered rapidly through pathways, such as MyD88, that do not require specific B cell recognition and would therefore afford some level of “specific” antibody protection prior to the induction of BCR-mediated antibody production. Supporting this concept, XID, MD4, and OVA-BCR mice produce rotavirus-specific antibody and clear an initial rotavirus infection with normal kinetics (Fig. S4).

In summary, we demonstrate that Cd11c^+^ dendritic cell expression of MyD88 plays an important role in the ability of rotavirus to induce *in vitro* B cell activation, Peyer's patch B cell activation, and early production of rotavirus-specific antibody after viral exposure *in vivo*. Our finding that rotavirus cannot induce activation of purified B cells *in vitro* supports the conclusions of others that a non-T cell type is a critical aspect of the B cell response pathway during rotavirus infection. Future studies will be necessary to determine the relationship among type I interferon receptor signaling, toll-like receptor signaling, and class switch recombination to IgA in the context of an intestinal viral infection that induces T cell-independent IgA.

## MATERIALS AND METHODS

### Mice

Male and female adult mice were used. Mice expressing a B cell receptor specific for OVA (C.B17 non-transgenic littermate control) were kindly provided by Dr. Lynn Dustin (51). Mice lacking MyD88 (C57BL/6 littermate control) were kindly provided by Dr. Shizuo Akira (52). TACI^−/−^ and APRIL^−/−^ mice (C57BL/6 control) were kindly provided by Dr. Raif Geha (53, 54). BAFF^−/−^ mice (C57BL/6 littermate control) were kindly provided by Dr. William Stohl (55). IFNaR^−/−^ (C57BL/6 littermate control) were kindly provided by Dr. Brenden Lee (56). Mice were bred in-house at Baylor College of Medicine, Houston, TX. For the following strains, breeders were obtained from Jackson Laboratories (Bar Harbor, ME) or Taconic Biosciences (Germantown, NY) and bred in-house: B6.FVB-Tg (Itgax-DTR/EGFP) (57), Lan/J (FVB/N non-transgenic littermate control) (20), MHCII (C57BL/6 control) (57), TIRAP (C57BL/6 littermate control) (41), TRIF (C57BL/6 littermate control) (58), and MD4 (C57BL/6 non-transgenic littermate control) (59). CD-1, BALB/c, and C57BL/6 mice for Fig. S3 were obtained from Charles River Laboratories (Wilmington, MA). CBA/CaHN-Btk(xid)/J (CBA/CaJ control) (60), CD40^−/−^ (C57/BL6 control) (61, 62), and CD40L^−/−^ (C57BL/6 control) (62) were obtained from Jackson Laboratories directly and used in experiments. Mice were housed in microisolator cages and fed *ad libitum*. All animals were cared for under veterinary surveillance.

### Flow cytometry

Data were acquired using a Coulter EPICS XL-MCL (Beckman Coulter, Hialeah, FL) flow cytometer. We collected 10,000–50,000 events for each sample. Forward scatter versus side scatter was used along with propidium iodide staining to gate on viable cells and exclude dead cells. Doublet discrimination (forward scatter area versus forward scatter height) was used to exclude doublets. Single fluorescent controls were used to determine true positive populations and to correct for spectral overlap. Unstained controls were used to assess autofluorescence and to define negative populations. Where appropriate, known treatments that induce or reduce B cell activation were included as biological controls. BD software (FACSDiva Version 6.1) was used to analyze and quantify the amount of fluorescence. Boolean gating strategies (co-expression of CD69 and lymphocyte marker) were used to assess co-expression of markers.

### Rotavirus infection

Mice were orally inoculated with 10^3^–10^5^ ID_50_ of the EC_wt_ strain of rotavirus (originally obtained from Harry Greenberg, Stanford University Medical School, Palo Alto, CA) in 100 µL of phosphate-buffered saline (PBS) as described previously (3). The ID_50_ for each mouse strain was determined as described previously (63, 64). Briefly, each background strain was inoculated with various dilutions of the virus stock and the highest dilution that caused shedding of the virus in the fecal pellets of 50% of the animals was considered the ID_50_. Fecal pellets were analyzed for the presence of rotavirus antigens by enzyme-linked immunosorbent assay (ELISA) to confirm infection (8, 63). Total systemic and fecal levels of rotavirus-specific IgG, IgM, and IgA antibody titers were measured by ELISA as previously reported (8, 63).

### *In vivo* dendritic cell depletion

To deplete dendritic cells, we administered 6- to 10-week-old male and female offspring expressing the CD11c-DTR transgene 0.4 ng/g body weight of diphtheria toxin (Sigma-Aldrich, St. Louis, MO) in sterile water or water alone intraperitoneally 1 day prior to, and 2 days after rotavirus inoculation as previously described (20). All mice were group housed (five per cage) in microisolator cages and fed *ad libitum*. Prior to infection with rotavirus, deletion of CD11c^+^ cells was confirmed by the lack of GFP expression in PBMCs.

### Peyer's patch cell activation

Two days following virus inoculation, single cell suspensions were prepared from all visible Peyer's patches of uninfected and infected mice. Single cell suspensions were prepared using mechanical disruption and pressing through a 70 micron pore-sized cell strainer (Fisher Scientific, Pittsburgh, PA) followed by treatment with ACK lysing buffer (BioWhittaker, Walkersville, MD) to induce red blood cell lysis (19). Leukocyte activation was assessed by incubating separate aliquots of two million cells for 30 minutes at room temperature with fluorescein isothiocyanate-labeled anti-CD4, CD8, CD19, CD11c, CD11b, or CD138 (each at a concentration of 0.06 µg/10^6^ cells obtained from BD Pharmingen, San Diego, CA) in combination with phycoerythrin-labeled CD69 (0.06 µg/10^6^ cells obtained from BD Pharmingen) followed by fixation in 4% paraformaldehyde (Fischer Scientific, Pittsburgh, PA). The percentage of activated leukocytes out of total leukocytes was determined for each sample. Each experiment evaluated responses of a minimum of three mice and was repeated at least twice.

### Fragment culture

Local production of rotavirus-specific antibody in the Peyer's patches was measured by ELISA using the fragment culture method as described previously (8). Briefly, following removal and washing, we incubated the Peyer's patches in 400 µL of GALT medium for 4 days. The amount of total rotavirus-specific antibody (IgG, IgA, and IgM) secreted by the Peyer's patches was assessed by ELISA. The experiments were performed a minimum of twice with three to five animals per group.

### *In vitro* B cell activation and flow cytometry

Single cell suspensions were prepared from spleens of CD-1 naïve mice and treated and analyzed as described previously (19). Spleens were used instead of Peyer's patches to obtain more cells since there appeared to be no difference between dendritic and B cell activation between the two tissues/organs (19). Briefly, two million splenic cells in 100 µL of medium were isolated and incubated overnight with 0.01 µg/µL of purified rhesus rotavirus (RRV) (65) grown and purified as previously described (19) from an isolate originally obtained from Dr. Harry Greenberg. As controls, separate wells of cells were treated overnight with either media alone (RPMI 1640 Medium [Lonza, Basel, Switzerland] supplemented with 10% FBS [Sigma-Aldrich, St. Louis, MO], 200 mM l-glutamine, 10,000 U/mL penicillin and streptomycin, 50 mM beta-mercaptoethanol, and 5% NCTC-109 medium [Gibco-BRL, Rockville, MD]), 0.01 µg/µL of *Escherichia coli* serotype 0111:B4 lipopolysaccharide (LPS, Sigma Chemical Company, St. Louis, MO), 0.01 µg/µL of phorbol myristate acetate (PMA; Sigma Chemical Co, St. Louis, MO), or (i.v.) 0.01 µg/µL boiled RRV (19). B cell activation was assessed by incubation of treated suspensions for 30 minutes at room temperature with phycoerythrin-labeled CD69 (0.03 µg/10^6^ cells) and fluorescein isothiocyanate-labeled CD19 (0.06 µg/10^6^ cells) both obtained from BD Pharmingen (San Diego, CA) followed by fixation in 4% paraformaldehyde (Fischer Scientific) and analysis using flow cytometry. The percentage of activated B lymphocytes out of total B lymphocytes was calculated for each sample. Each experiment contained a minimum of three spleens and was repeated at least twice, and significant differences between groups were determined by Mann-Whitney U.

### Cell selection

Single cell suspensions of two million cells were prepared from spleens of naïve mice using mechanical disruption followed by red blood cell lysis (19). CD90^+^, CD11c^+^, CD11b^+^, and CD4^+^ cells were positively selected using the EasySep Mouse CD11c Positive Selection Kit, the EasySep Mouse CD11b Positive Selection Kit, and the EasySep Mouse CD4 Positive Selection Kit (StemCell Technologies, Vancouver, BC, Canada) and the Miltenyi pan DC microbeads or the Miltenyi CD90 microbeads (Miltenyi Biotec, Inc., Auburn, CA), respectively, following the manufacturer's instructions. B cells were negatively selected using either the EasySep Mouse B Cell Enrichment Kit (StemCell Technologies) or the Miltenyi B Cell Isolation Kit (Miltenyi Biotec, Inc.) following the manufacturer's instructions. Purity was assessed by flow cytometry as we have described previously (19) and generally was between 90% and 95%. Untouched cells (presort), selected cells (sort), or selected cells recombined with removed cells (add back) were treated overnight as described above.

### Interferon ELISA

ELISAs were performed on the supernatants from Peyer's patch cell suspensions from either WT or MyD88^−/−^ mice treated with either media alone, RRV, or boiled RRV overnight. Supernatants were collected, and interferon alpha was measured using the Verikine Mouse Interferon Alpha ELISA kit from PBL InterferonSource (Piscataway, NJ).

### Statistics

Differences between groups were calculated using Mann-Whitney U two-tailed significance using SPSS statistical software version 17.0 (IBM, Armonk, NY). A *P* value < 0.05 was considered significant. Each experiment was performed a minimum of two independent times, and a representative experiment is shown.

## Data Availability

All data, metadata, and methods used to reach the conclusions in the paper and any additional data required to replicate the study findings are available upon request.

## SUPPLEMENTAL MATERIAL

Supplemental material jvi.00653-25-s0001.pdf Figures S1 to S6.

ASM does not own the copyrights to Supplemental Material that may be linked to, or accessed through, an article. The authors have granted ASM a non-exclusive, world-wide license to publish the Supplemental Material files. Please contact the corresponding author directly for reuse.
