# Supplementary material for: Dendritic cell expression of MyD88 is required for rotavirus-induced B cell activation
Source: J Virol. 2025 Apr 30;99(5):e00653-25. doi: 10.1128/jvi.00653-25 (PMC12090804; doi:10.1128/jvi.00653-25)
Supplement: Supplemental material — Figures S1 to S6. [file jvi.00653-25-s0001.pdf]

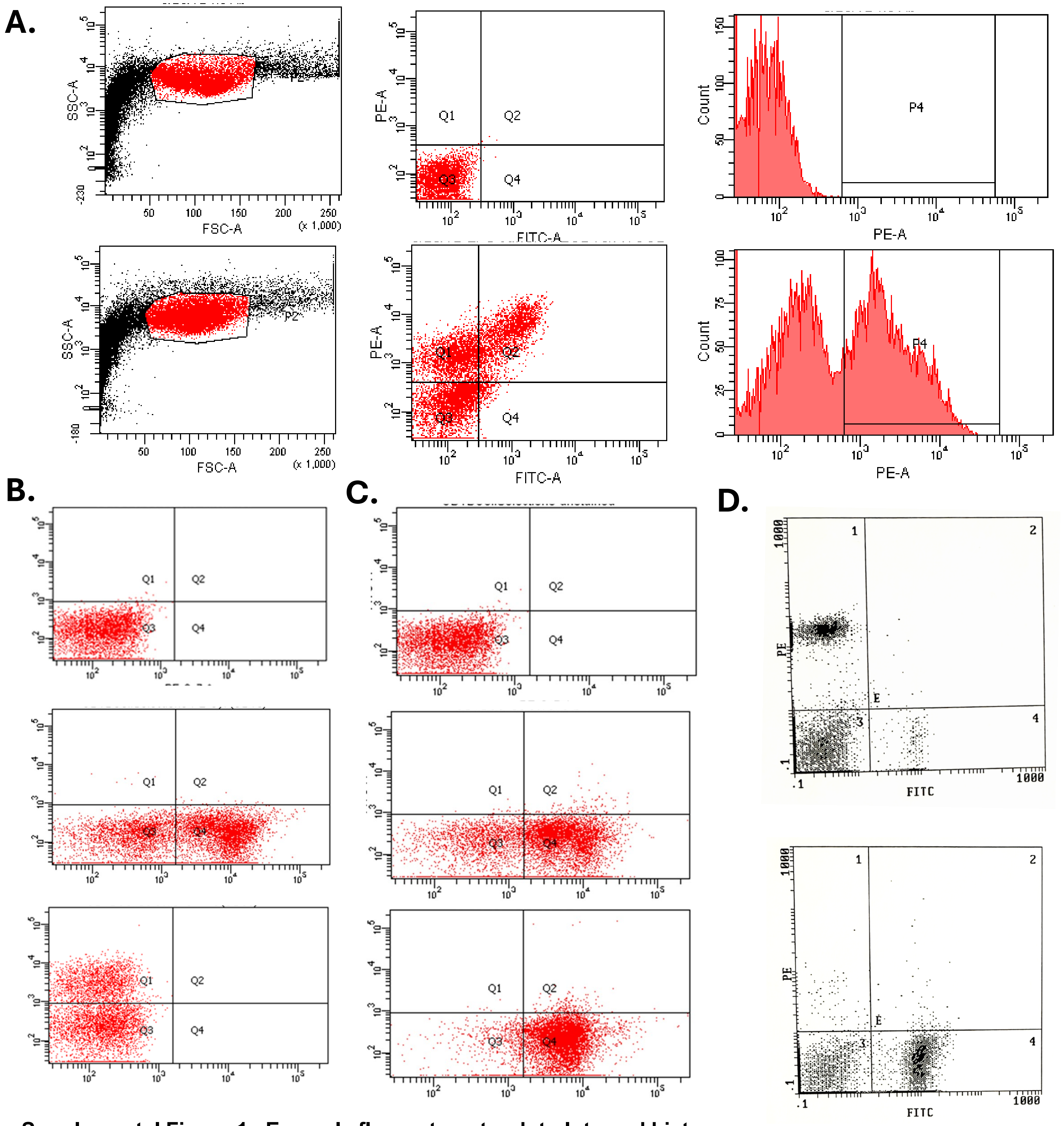

Supplemental Figure 1. Example flow cytometry dot plots and histogram

A. Peyer's patch single cell suspension were incubated with antibodies against CD19 (FITC) and CD69 (PE). Forward scatter (FSC-A) and side scatter (SSC-A) were used to gate on viable cells and to exclude dead cells. Unstained cells were used to set gates (top row). Boolean gating was used to quantify the numbers of total FITC+ cells and the numbers of FITC+/PE+ cells. Percentage activated cells was calculated by dividing the number of activated cells (FITC+/PE+) by the total number of FITC+ cells. The same strategy was used for CD19, CD138, CD4, CD8, CD11b, and CD11c using individual samples from the same Peyer's patch cell suspension. B. Splenic or Peyer's patch single cell suspensions were used to adjust compensation. Top, unstained. Middle, PE-CD69 antibody only. Bottom, FITC-CD19 antibody only. C. Purity of negatively selected B cells stained with FITC-CD19. Top, unstained. Middle, unsorted. Bottom, CD19 negative selection. D. Cell suspensions depleted of CD90 cells and stained with FITC-CD19 and PE-CD90. Top, unsorted. Bottom, depleted of CD90.

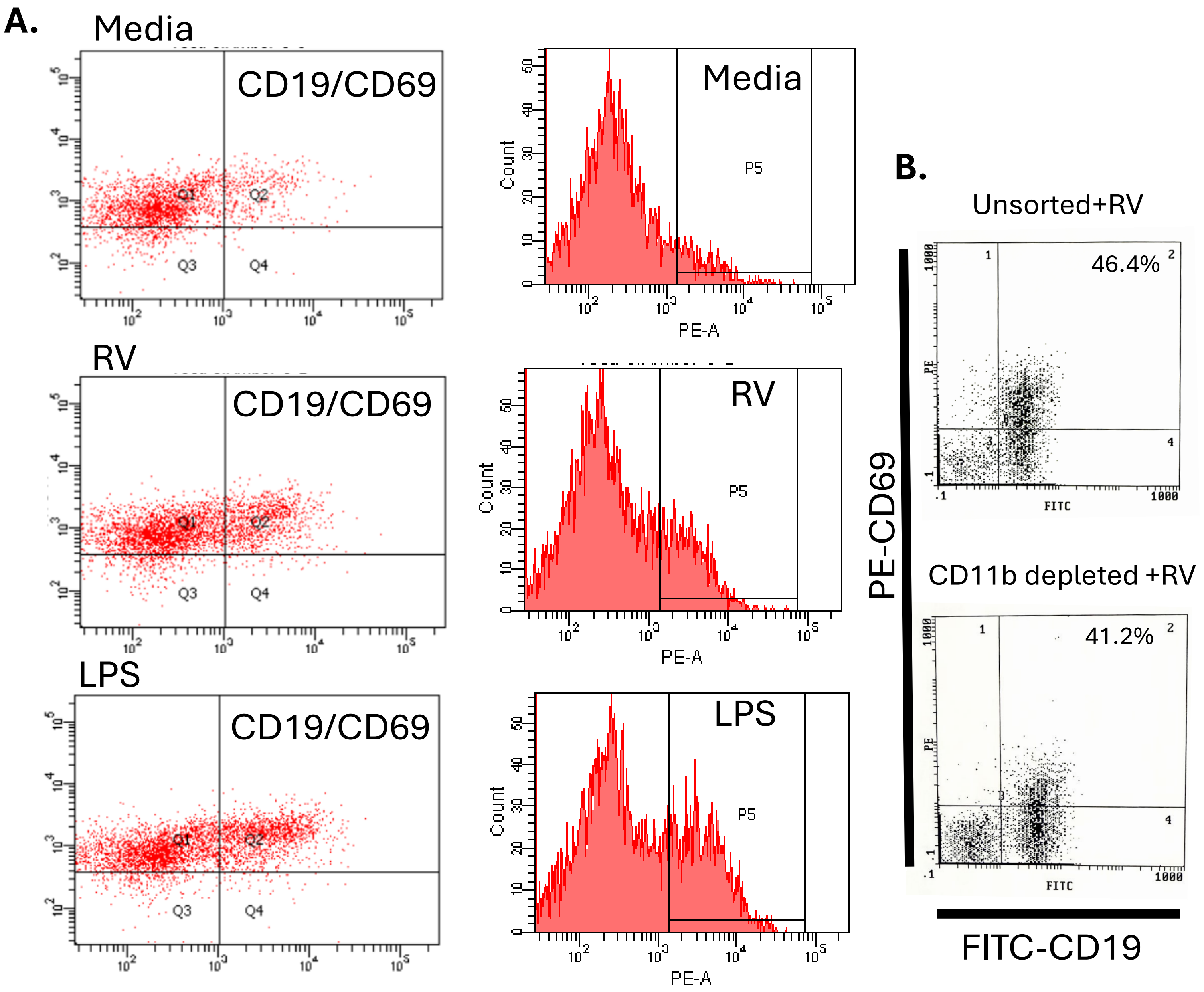

Supplemental Figure 2. Example Quantification of activated B cells.
A. Splenic single cell suspensions were treated with either media (top), rotavirus (middle), or LPS (bottom) followed by assessment of B cell activation by expression of CD69 on the B cell population using fluorescently labeled antibodies and flow cytometry. CD19+ cells (FITC) were gated upon and the number of cells expressing CD69+ (PE) B cells was quantified using the histogram. B. Splenic single cell suspensions were depleted of CD11b+ cells using magnetic beads. Activation was assessed by quantifying FITC-CD19/PE-CD69.

A. CD-1

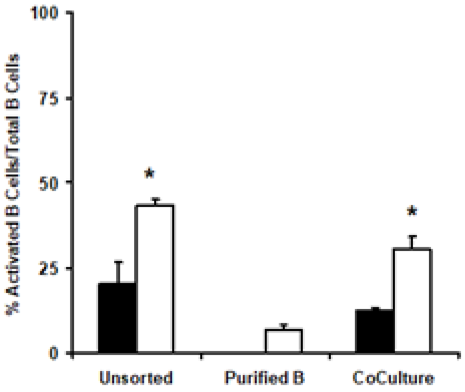

B. C57BL/6

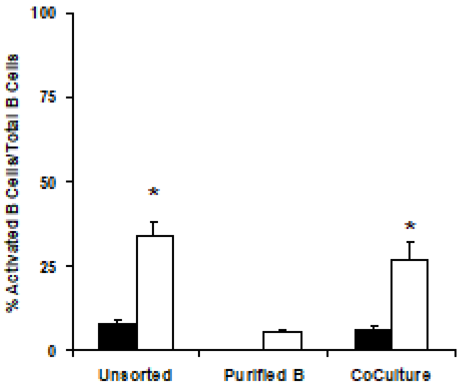

C. BALB/c

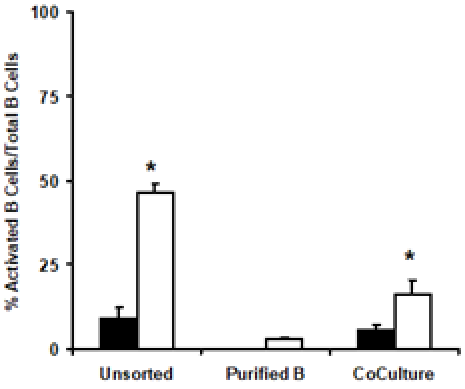

Supplemental Figure 3. Dendritic cells are sufficient to activate B cells following rotavirus treatment.
A-C, Splenic single cell suspensions were treated with either media (black bars) or rotavirus (white bars) followed by assessment of B cell activation by expression of CD69 on the B cell population using fluorescently labeled antibodies and flow cytometry. B cells were examined prior to manipulation (Unsorted) or negatively selected and either co-cultured alone (Purified B) or cultured with positively selected $CD11c^{+}$ dendritic cells (CoCulture). A, CD-1 mice, B, C57BL/6 mice, C, BALB/c mice. Each bar represents the mean number of activated cells out of total B cells from 3 individual mice + SD. *, $p<0.05$ by Mann Whitney U compared to purified B cells treated with rotavirus. This experiment was repeated three independent times with a representative experiment shown.

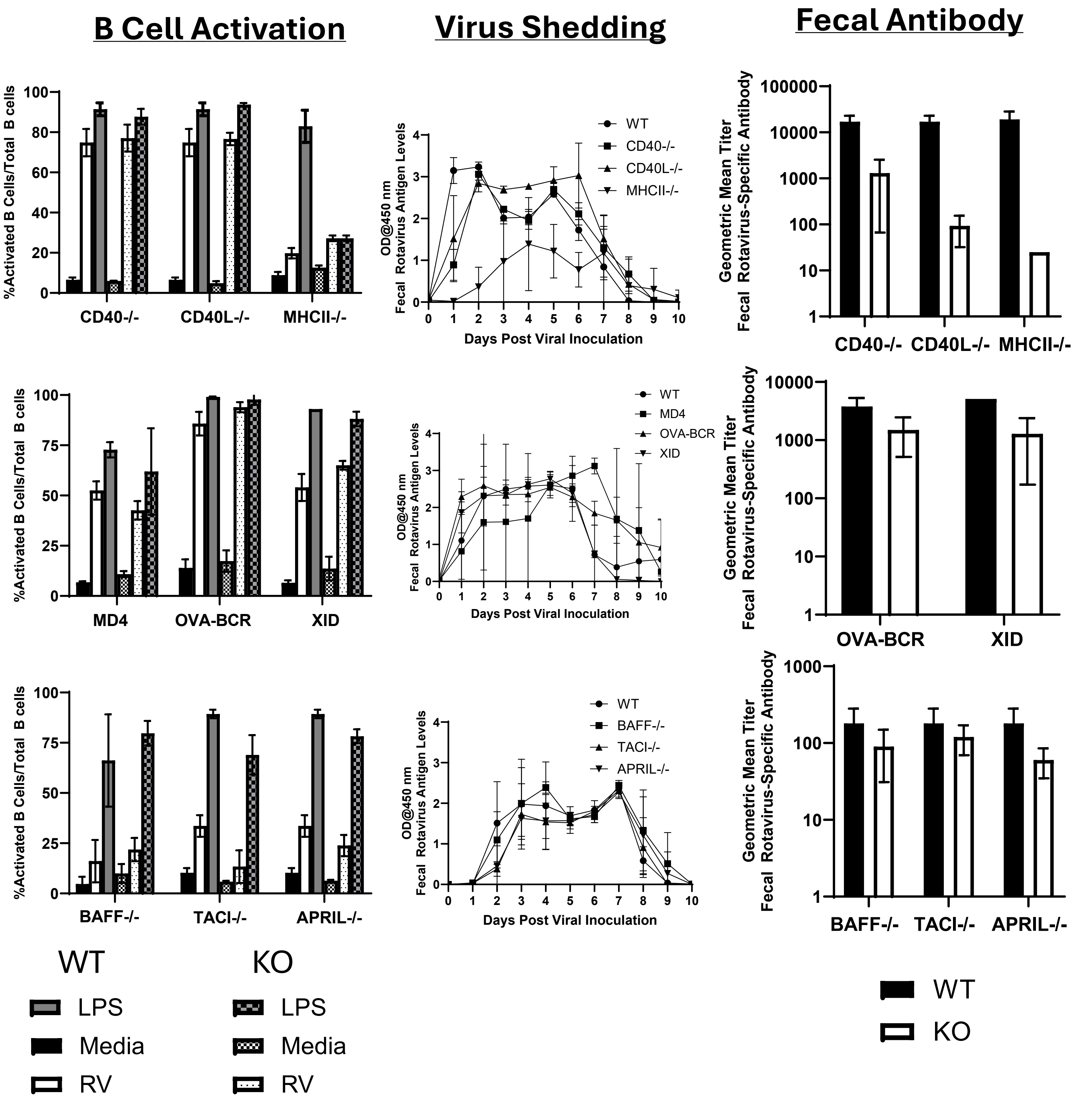

Supplemental Figure 4. B cells in mice lacking specific signaling molecules can still be activated following rotavirus treatment, mice clear rotavirus infections and produce fecal rotavirus specific antibody.

Left panels: Splenic cells from the indicated knockout mice were stimulated overnight with media, 0.01 ug/ul purified rotavirus (RV), or 0.01 ug/ul LPS, and the percent of $CD19^+$ cells that expressed CD69 out of the total $CD19^+$ population was determined by flow cytometry for each mouse. Mean ± SD (n=3-7) are shown. Middle panels: Mice were infected with 10^3-10^5 SD50 of EC_{wt} and fecal pellets collected daily and analyzed by ELISA for rotavirus antigens. Right panels: Rotavirus specific antibody (IgA, IgG, and IgM) was quantified by ELISA and geometric mean titers were determined 21 days following rotavirus infection. Each bar or point represents 3-6 individual animals and each experiment was performed at least two independent times with a representative experiment shown.

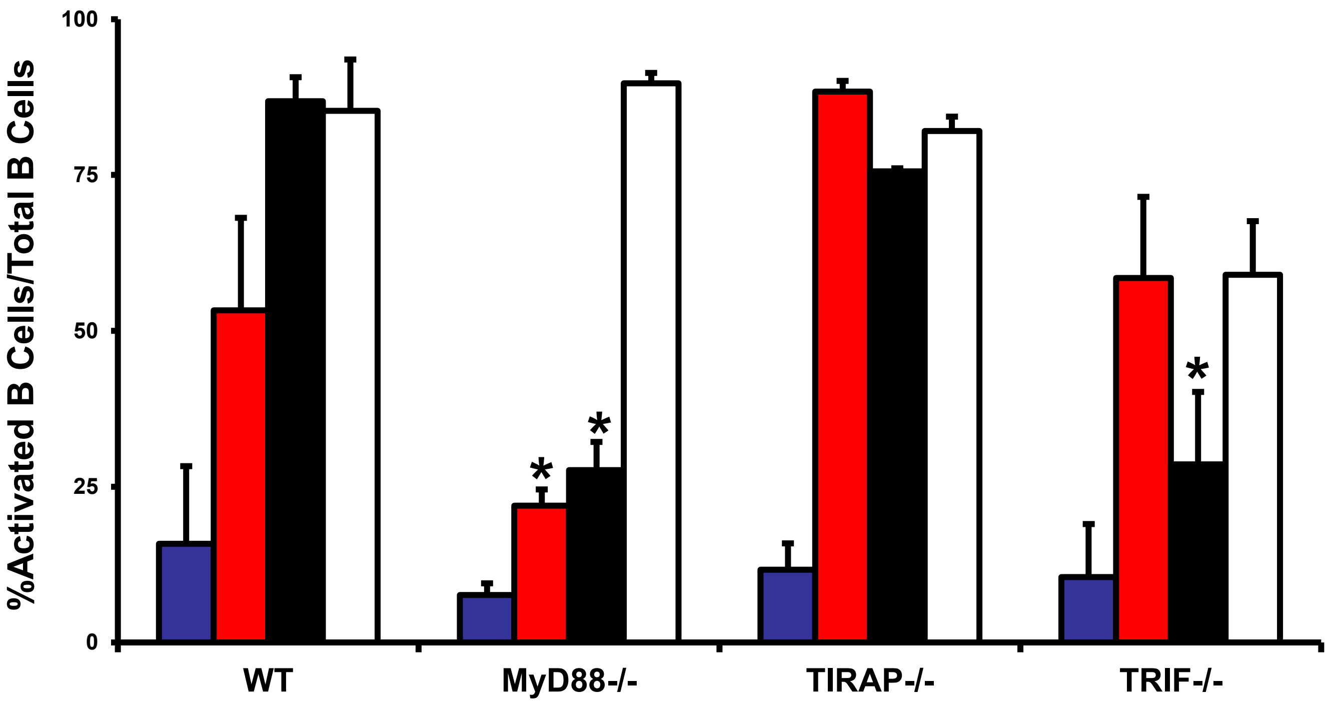

Supplemental Figure 5. B cells in mice lacking toll-like receptor pathway signaling molecules can still be activated following rotavirus treatment.
Splenic cells from the indicated knockout mice were stimulated overnight with media (blue bar), 0.01 ug/ul purified rotavirus (red bar), 0.01 ug/ul LPS (black bar), or 0.01 ug/ul PMA (white bar) and the percent of $CD19^+$ cells that expressed CD69 out of the total $CD19^+$ population was determined by flow cytometry for each mouse. Mean ± SD (n=3-7) are shown. *, $p<0.05$ compared to media treated group. This experiment was repeated at least three independent times, and a representative experiment is shown.

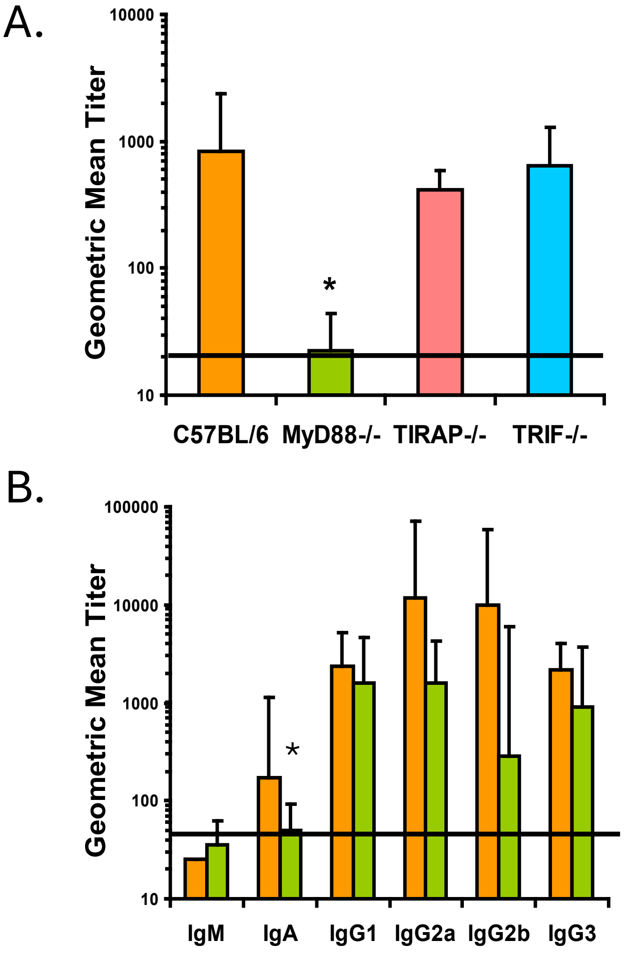

Supplemental Figure 6. MyD88, but not TIRAP or TRIF expression is critical for rotavirus specific IgA production.

C57BL/6, MyD88-/-, TIRAP-/- and TRIF-/- mice were inoculated with rotavirus. A. Ten days after inoculation, fecal samples were analyzed for the presence of rotavirus-specific IgA by ELISA. Geometric mean titer ± SD (n=6-8 mice/group) was calculated for each group. *, $p<0.05$ compared to all other groups. Horizontal line indicates positive cutoff value. B. Serum samples from rotavirus-infected C57BL/6 (orange bar) or MyD88-/- (green bar) mice were analyzed after 21 days by ELISA for rotavirus specific antibody isotypes as indicated. Geometric mean titer ± SD (n=6-8 mice/group) was calculated for each group. *, $p<0.05$ compared to C57BL/6 group. Horizontal line indicates positive cutoff value. This experiment was performed at least twice, and a representative experiment is shown.
